# Low-field NMR investigations on dynamics of crude oil confined into nanoporous silica rods and white powder

**DOI:** 10.3389/fchem.2023.1087474

**Published:** 2023-01-26

**Authors:** Salim Ok

**Affiliations:** Petroleum Research Center, Kuwait Institute for Scientific Research, Kuwait City, Kuwait

**Keywords:** crude oil, confined state, relaxation, low-field NMR, nanoporous silica

## Abstract

In the present study, to mimic the natural confinement of crude oils, model experiments are conducted with crude oils having different physical properties and maltenes of parent crude oils without asphaltenes confined into engineered nanoporous silica rods with pore diameters of 2.5 and 10.0 nm and white powdered nanoporous silica with pore diameters of 2.5 and 4.0 nm. This will help with suggesting potential treatments for enhancing crude oil recovery. Low-field nuclear magnetic resonance (LF-NMR) relaxometry has been applied to achieve this goal. The nanoporous proxies resemble real-life nanoporous rocks of reservoirs. The dynamics of confined crude oils with different ^o^API gravity deviate from bulk dynamics, and deviation changes depending on the ^o^API gravity. This suggests that treatments must be decided appropriately before crude oil production. Similar treatments could be applied for light and medium-heavy crude oils. Mathematical analysis of NMR relaxation curves of confined crude oils with different fractions of SARA (saturates, aromatics, resins, asphaltenes) indicates that the conventional SARA approach needs a better definition for the confined state of matter. The NMR relaxation behavior of confined maltenes shows that resin molecules might act like saturates in natural confinement with various scale pores from nano to micro and even macro, or aromatics might show resin-like behaviors. Confinement of brine and a light crude oil into white powdered nanoporous silica proxies demonstrates that brine could be utilized along with some additives such as nanoparticles for oil recovery. Therefore, these issues must be evaluated in deciding the proper treatments for crude oil production.

## 1 Introduction

In Earth’s crust, a significant percentage of crude oils remains still in the nanopores of reservoir rocks after flooding (Gautam et al., 2017; Taborda et al., 2017). The accessible porosity within the rocks has various lengths (d as pore diameter or fracture aperture) such as micro-, meso-, and macroporous ranges (d < 2.0 nm, 2.0 < *d* < 50 nm, and *d* > 50 nm, respectively, as defined by IUPAC). These nanopores manage the permeability of reservoirs, and the fraction of nanopores with diameters in the range from 0.5 to 100.0 nm is approximately 80% of the rock porosity of the reservoirs (de Almeida and Mirana, 2016; Wang et al., 2018). Therefore, it is essential to gain insight into the dynamics of the crude oils in the nanopores interconnecting the large pores so that novel approaches for governing the flow of crude oils under confinement could be designed for potential applications in enhanced and improved oil recovery areas.

One of the common ways to explore the confined fluids experimentally is to prepare model systems at the laboratory scale followed by measurements mimicking the reservoir conditions, for example, in crude oils (Cole et al., 2013). Certain parameters, such as the dimension, profile, distribution, and interconnectedness of confined geometries, the interaction between fluid molecules, and the interaction between fluids of interest and the surface of the solid govern and control how fluids flow and behave in confined geometry (Ok et al., 2021). The flowing fluids, reactants, and products of intrapore zones move into and through these nano-environments. During this flow, various processes, such as wetting, ultimate adsorbing, interaction, and even reaction, happen on the solid surfaces (Gautam et al., 2017; Ok et al., 2021). Moreover, the effects of high temperature and/or pressure common to Earth sciences can significantly vary the dynamics of fluid behavior on wetted surfaces or confined geometries. Due to the complexity of C-H-O fluids, such as crude oil, a quantitative clarification of confined fluids interactions of confined fluids with solids is needed. Most of the time, the attitudes of fluids deviate in the confined state at solid interphases compared to the bulk behavior. These deviations are influenced by the size, shape, and topology of confinement and by the competition of fluid-fluid and fluid-matrix interactions as getting closer to the outermost layers of the matrix (Gelb et al., 1999; Cole et al., 2004; Vogel, 2010). The deviations are reflected in different physical properties, including melting temperature and the dynamical attitudes of the molecules. Basic understanding requires a detailed description of each of these confinement effects.

Investigating the fluid behavior under confinement has two crucial aspects: detailed characterization of the nanoporous host matrix system at first and then deviation degree of confined fluid attitude from that of the bulk. Among different methods, low-field nuclear magnetic resonance (LF-NMR) is one of the most robust ones in studying confined fluids because of being non-destructive, a relatively more facile method of sample preparation, and easy to operate (Liebscher and Heinrich, 2007; Dvoyashkin and Filippov, 2018). LF-NMR has been applied in the petroleum industry since the mid-1960s (da Silva et al., 2015) and allows studying translational and rotational mobility of confined molecules. Fluids explored under a confined state by NMR include methane, methanol, and water-type small molecules (Ok et al., 2017; Ok et al., 2020). These molecules and their similar versions were confined into various porous systems such as nanoporous MCM-41 (Xu et al., 2007), mesoporous Vycor glass (Dvoyaskin et al., 2007), and nanoporous silica rods (Ok et al., 2020).

The longitudinal (T_1_) and transverse (T_2_) magnetization relaxation times are frequently measured to study the dynamics of confined fluids by LF NMR relaxometry. The T_1_ and T_2_ magnetizations are the magnetization components parallel and perpendicular to the external magnetic field B_0_, respectively. The time needed to align the nuclei along the same direction of the applied external magnetic field is defined as T_1_ (Freedman and Heaton, 2004), while T_2_ explains the tendency of phase-coherent nuclei or spins present in a homogeneous external field to give up their coherence (Westphal et al., 2005). T_1_ is controlled by energy, while entropy governs T_2_. Determining T_1_ and T_2_ are classical ways to explore molecular reorientations (Vogel, 2010).

T_1_ and T_2_ relaxations could be subjected to mathematical inversion processes to obtain distributions. The T_1_ distributions reflect the complex composition of different systems, such as crude oils and the distribution of pore sizes in sedimentary rocks (Freedman and Heaton, 2004). T_1_ distributions estimate pore-size distributions in rock samples (Song and Kausik, 2012; Walbrecker and Behroozmand, 2012). As in crude oil, T_2_ decaying curves correlate to the viscosity of different complex mixtures (Freedman and Heaton, 2004). When samples of saturated porous media are measured, the amplitude of the T_2_ measurements is directly proportional to porosity, and the decay rate is related to the pore size, the fluid type, and its viscosity in the pore space. Short T_2_ times generally indicate tiny pores with large surface-to-volume ratios and low permeability. Conversely, longer T_2_ times indicate larger pores with higher permeability (Anovitz and Cole, 2015). Pore size distribution in sedimentary rocks varies depending on several factors, such as formation events and chemical processes. Therefore, the pore size distribution might change from nanometer scale to micrometer from one reservoir to another. However, it is essential to note that pores are classified as macropores with diameters longer than 50 nm, mesopores with diameters between 2 and 50 nm, and micropores with diameters smaller than 2 nm (Kashif et al., 2019). For example, Lyu et al. (2018) studied the pore size distribution of tight sandstone by LF-NMR of subsurface probes from 3 nm to 100 μm.

The contribution of the confined fluid to the NMR relaxation, according to Coates et al. (1999), relies on bulk, surface, and molecular diffusion characteristics:

$$\frac{1}{T_{1}}=\frac{1}{T_{1bulk}}+\frac{1}{T_{1surface}}$$ (1)

$$\frac{1}{T_{2apparent}}=\frac{1}{T_{2bulk}}+\frac{1}{T_{2surface}}+\frac{1}{T_{2diffusion}}$$ (2)

T_*i*bulk_ relaxation times of the confined fluid would be the values as the relaxation values would be measured with ignorable surface effects. On the contrary, T_*i*surface_ relaxation times of the confined fluids result from the surface relaxation. T_2diffusion_ relies on the molecular diffusion coefficient, D, and the magnetic field gradient, G. The D values of confined fluids depend on pore geometry and the properties of pore surfaces (Medina-Rodriguez et al., 2020). In confined spaces and volumes, interacting with surfaces results in competition between the liquid-liquid and surface-liquid interactions.

In the present study, dynamics of crude oils with four major components of “saturates, aromatics, resins, and asphaltenes (SARA)” and their corresponding maltenes, the portion of crude oils without asphaltenes are studied upon confining into nanoporous silica rods and white powders. These engineered nanoporous proxies are selected because these proxies affect different physical properties of confined fluids and provide a similar medium resembling real-life nanoporous rocks of reservoirs. The LF-NMR experiments are conducted to reveal the dynamics of confined crude oils to understand the behavior of crude oils in the nanopores of reservoir rocks. The aim is to mimic the natural confinement of crude oils inside reservoirs with all the possible pore dimensions. This will help explore crude oil’s dynamic behaviors and interactions with different pores representing the natural rock core systems.

## 2 Materials and methods

### 2.1 Samples

Both toluene and n-heptane (Analytical reagent grade) were purchased from Fisher Scientific. The solvents were used without further purification. Sodium chloride (NaCl), calcium chloride (CaCl_2_), and magnesium chloride (MgCl_2_) were purchased from Sigma Aldrich and used without purification for preparing brine solution in deionized water. Asphaltene samples were extracted from different crude oils by standard methods described elsewhere, following the standard procedure of IP143 (Table 1), and hence maltenes are obtained (Mullins, 2011; Majumdar et al., 2013). The physical properties of crude oils and their corresponding maltenes confined into engineered proxies are given below (Table 1) (Mullins, 2011; Majumdar et al., 2013). Mesoporous silica (200 nm average particle size and 4 nm average pore diameter) was purchased from Sigma-Aldrich, while mesoporous silica-2.5 nm was synthesized at the laboratory (Liu et al., 2006; Liu et al., 2013). Silica-2.5 nm was synthesized using only different carbon chain length surfactants or hydrothermal treatments (Liu et al., 2013). Then silica-2.5 nm (powder) was treated with the second hydrothermal, thus having an even stronger structure and better hydrolysis resistance. More details on the synthesis of silica-2.5 nm (powder) are given in another report by Liu et al. (2006). The silica porous monolith samples were purchased from Particle Solutions, LLC (Alachua, FL).

**TABLE 1:** The physical properties of crude oils and their corresponding maltenes confined to nanoporous silica rods and nanoporous silica powder.

			Crude oils			Maltenes		
Characteristic	Unit	Test method	1	2	3	4	5	6
Density @ 25°C	g/cm^3^	ASTM D5002	0.97318	0.87024	0.83707	0.98078	0.96046	0.91272
Specific gravity		ASTM D5002	0.98043	0.87813	0.84520	0.98799	0.96779	0.92033
API	°	ASTM D5002	12.82	29.64	35.92	11.72	14.71	22.25
Kinematic viscosity @ 40°C	cSt	ASTM D445	475.14	12.10	4.07	891.23	408.47	63.03
Total acid number	mgKOH/g	ASTM D664	1.25	0.56	0.97	2.35	1.28	0.79
Sulfur content	wt%	ASTM D4294	4.84	2.82	0.91	5.12	4.13	2.15
Micro carbon residue	wt%	ASTM D4530	12.00	6.53	2.44	10.34	8.06	4.41
Refractive index	nd at 20°C	ASTM D1218	1.55464	1.50218	1.47782	1.55700	1.54579	1.51507

ASTM, American Society for Testing and Materials; API, American Petroleum Institute.

### 2.2 Basic characterization

Measurements of BET surface areas, pore volumes, and diameters from the engineered silica rods (Table 2) were obtained with nitrogen adsorption and desorption at 77 K utilizing a Micromeritics ASAP 2020 surface area and porosity analyzer. Before the adsorption−desorption measurements, the samples were degassed at 150°C for 180 min under a vacuum pressure of 10 μm Hg.

**TABLE 2:** Surface area, pore volume, and average pore size of the engineered silica rods.

Sample	BET surface area	Total pore volume	Adsorption average pore width (Å)
Silica rod-2.5 nm	494.5 m^2^/g	0.63 cm^3^/g	49.7
Silica rod-10 nm	118.5 m^2^/g	0.30 cm^3^/g	101.8

The white powder mesoporous silica particles with a 4 nm pore diameter have a spherical morphology, and the sizes of the particles are uniform (reported by the manufacturer as 200 nm particle size). TEM imaging of this material revealed particles of approximately 4 nm diameter with an ordered array of pores, verified with BET-BJH analysis and matching the specifications provided by the manufacturer (Ok et al., 2017). Silica-4.0 nm and silica-2.5 nm (white powders) have surface areas of 597 and 1,167 m^2^/g, respectively. These two white powdered samples’ pore volumes are 1.04 and 0.97 cm^3^/g for silica-4.0 nm and silica-2.5 nm. The surface area to volume ratio for silica-4.0 nm is lower, with a value of 574, than that of silica-2.5 nm, with a value of 1,203 (Ok et al., 2020).

### 2.3 Sample preparation: Confinement of crude oils and maltenes into nanoporous silica rods and white powdered nanoporous silica

The nanoporous silica rods were kept in crude oils and maltenes for several weeks at 25°C for the complete filling of the nanopores. During the saturating process of the pores, the beakers or vials were sealed properly by several layers of parafilm to prevent the evaporation of lighter phases. After filling the silica rods’ nanopores, the outer surface of the silica rods was cleaned with a napkin to ensure that no crude oil or maltenes were left on the outer surface of the nanoporous silica rods.

To prepare the brine solution, 58.4 g NaCl, 111.0 g CaCl_2_, and 95.0 g MgCl_2_ were dissolved in 0.710 L deionized water at 25°C. The concentration of the brine is 250.000 ppm. Table 3 lists the solutions prepared. Hence, the surface area of nanoporous silica samples and the total amount of confined fluids (0.4 ml) are kept constant. This allows for comparing pore volumes’ effect on the confined fluids’ behavior. In addition, the mixtures were left at 25°C for at least 2 days for complete saturation of the nanopores before the low-field NMR measurements.

**TABLE 3:** Samples prepare by blending white powdered nanoproous silica-4.0 and silica-2.5 nm with either brine only, light crude oil only, or both fluids.

Silica sample	Brine	Light crude oil
200 mg silica-4.0 nm	0.4 ml	—
100 mg silica-2.5 nm	0.4 ml	—
200 mg silica-4.0 nm	—	0.4 ml
100 mg silica-2.5 nm	—	0.4 ml
200 mg silica-4.0 nm	0.08 ml	0.32 ml
100 mg silica-2.5 nm	0.08 ml	0.32 ml
200 mg silica-4.0 nm	0.12 ml	0.28 ml
100 mg silica-2.5 nm	0.12 ml	0.28 ml

Brine: Prepared using NaCl, CaCl2, and MgCl2 with a concentration of 250.000 ppm; Light crude oil: ^o^API gravity of 35.92 and density of 0.83707 g/cm3 at 25^°^C (light crude oil is sample 4 in Ok et al., (2019)).

### 2.4 Low-field NMR relaxometry measurements

T_2_ NMR measurements were performed on a Bruker Minispec mq20 NF Series instrument with a magnetic field strength of 0.47 T corresponding to a proton resonance frequency of 20 MHz at a magnet temperature of 40°C with Minispec software. The instrument was equipped with a 10 mm temperature-variable probe. Transverse magnetization relaxation (T_2_) was measured using the standard Carr-Purcell-Meiboom-Gill (CPMG) pulse sequence found in the Bruker library at 40°C. The T_2_ measurements were conducted with a time delay between 90° and 180° pulses (t) of 0.2 ms for confined crude oil and maltenes, while the delay time was 0.5 ms for bulk samples. The number of data points was adjusted for each sample before each T_2_ measurement, and T_2_ data were acquired with 1,024 scans, and the delay time was chosen long enough, 5*T_1_, to enable complete decay of the T_2_ signal. The repetition time between two subsequent scans was set to 5 s. The same parameters were utilized for confined crude oil inside nanopores of silica-4.0 nm and silica-2.5 nm (white powder).

In the present study, the three-exponential fitting analysis of the acquired T_2_ decays was performed in the Microcal Origin software using the following function for total NMR signal *y*:

$$\left(x\right)=\sum_{i=1}^{3}A_{i}\exp\left(-\frac{x}{T_{2\left(i\right)}}\right)$$ (3)

where *x* stands for the signal detection time, T_2(i)_ is the transverse relaxation time of the *i*-th component with respective amplitude A_i_. Fraction of each T_2_ was determined as (A_1_/(A_1_ + A_2_ + A_3_)) × 100. The T_2(1)_, T_2(2),_ and T_2(3)_ values were later used to determine correlations between crude oil properties and NMR relaxation behavior (Canan et al., 2022). The three-exponential fitting analysis of the acquired T_1_ decays was performed in the Microcal Origin software using the following function for total NMR signal *y*:

$$y\left(x\right)=\sum_{i=1}^{3}A_{i}\exp\left(\frac{x}{T_{1\left(i\right)}}\right)$$ (4)

where *x* stands for the signal detection time, T_1(i)_ is the transverse relaxation time of the *i*-th component with respective amplitude A_i_. Microcal Origin software gives T_2_ results with error bars.

The NMR transverse magnetization relaxation data were analyzed: a continuous distribution of T_2_ exponentials was fitted for all T_2_ decays using the CONTIN algorithm (Provencher, 1982). This analysis yielded a plot for the continuous T_2_ distribution.

## 3 Results and discussion

### 3.1 Dynamics of crude oils confined into nanoporous silica rods and nanoporous silica powder: T_2_ NMR results

**FIGURE 1:**
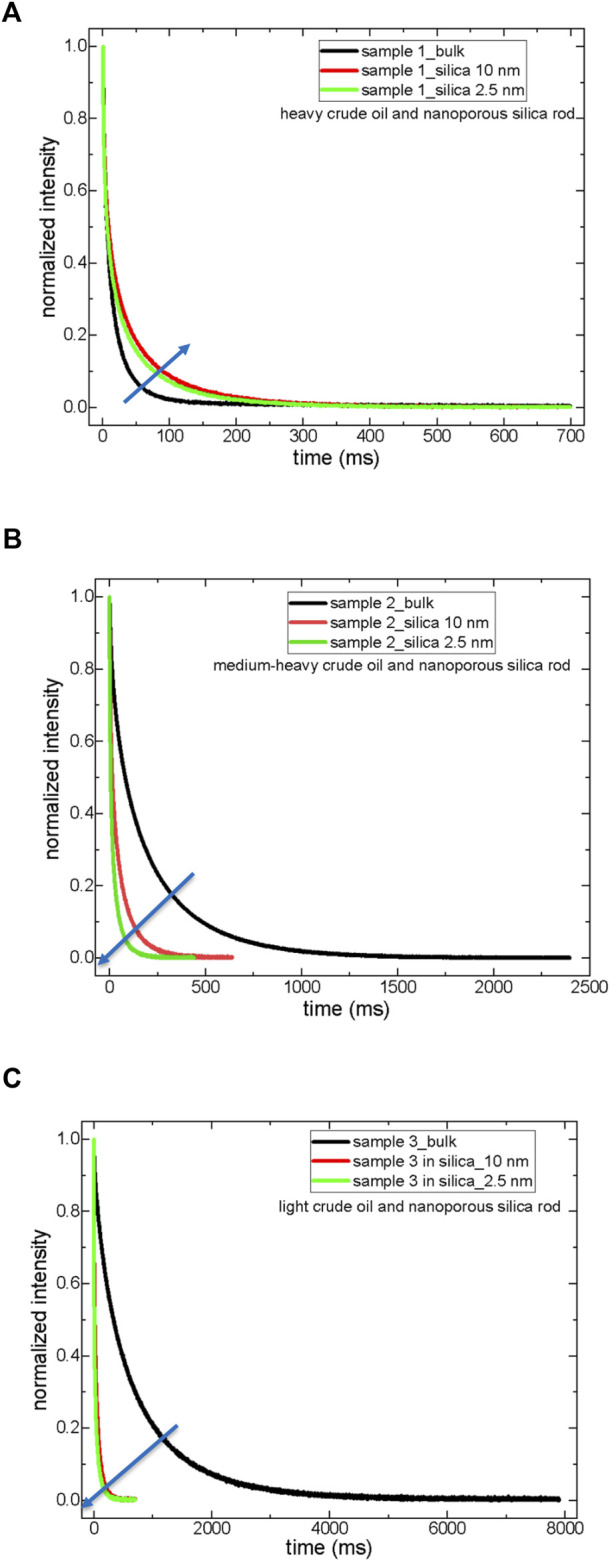
T_2_ relaxation curves of crude oils both in bulk and in a confined state; sample 1 **(A)**, sample 2 **(B)**, and sample 3 **(C)**.

Figure 1 exhibits the T_2_ relaxation curves of the samples both in bulk and confined states. At the same time, Table 4 summarizes the T_2_ relaxation values of crude oils and their corresponding maltenes in bulk and confined into nanoporous silica rods.

**TABLE 4:** T_2_ relaxation values of crude oils in bulk and confined state.

T_2_ relaxation	T_2_ (1) (ms)	T_2_ (2) (ms)	T_2_ (3) (ms)	A1%	A2%	A3%
Bulk crude oils						
1	3.2 ± 0.1	17.8 ± 0.3	80.0 ± 3.3	40.2	53.9	5.9
2	13.9 ± 0.2	88.7 ± 0.6	299.9 ± 0.5	16.5	33.0	50.5
3	78.7 ± 2.0	378.4 ± 4.4	1030.0 ± 4.0	10.6	39.4	50.0
Crude oils in silica 10 nm						
1	2.1 ± 0.0	17.9 ± 0.2	82.3 ± 0.4	36.2	36.2	27.6
2	2.6 ± 0.1	20.2 ± 0.3	81.9 ± 0.5	30.1	30.1	39.8
3	2.5 ± 0.1	19.6 ± 0.3	75.8 ± 0.3	24.3	29.1	46.6
Crude oils in silica 2.5 nm						
1	1.9 ± 0.0	15.3 ± 0.2	74.9 ± 0.4	40.2	34.6	25.2
2	1.2 ± 0.0	9.8 ± 0.2	43.0 ± 0.3	37.2	31.9	31.0
3	1.8 ± 0.0	15.6 ± 0.2	70.0 ± 0.4	34.9	33.0	32.1

The crude oils are the ones whose physical properties are briefed in Table 1. Sample 1: heavy crude oil, sample 2: medium heavy crude oil, sample 3: light crude oil.

First, T_2_ relaxation data of crude oils were treated with three component exponential decaying functions (see Eq. 3). The shortest T_2_ values, T_2(1)_, were assigned to the heaviest SARA components (asphaltenes and resins), while the longest T_2_ values, T_2(3)_, correspond to the saturated hydrocarbons, the lightest compounds in the sample. It was assumed (Volkov et al., 2021) that intermediate T_2(2)_ values characterize the aromatic compounds (heavier than the saturates but much lighter than asphaltenes and resins) in the samples. Second, in sample 1, a heavy crude oil, there is no significant deviation from bulk behavior when the sample 1 molecules are confined into nanoporous silica rod-10 nm. The confinement effect is slightly observed, as revealed by the shortening of T_2_ values when sample 1 molecules are confined into nanoporous silica rod-2.5 nm. Only T_2(1)_ assigned to asphaltenes and resins showed a decrease both in silica-10 nm and silica-2.5 nm, while both T_2(2)_ and T_2(3)_ values decreased only inside silica-2.5 nm.

In sample 2, a medium-heavy crude oil, T_2_ relaxation values decreased approximately 4-fold, confining into silica-10 nm and almost 7.5-fold inside silica-2.5 nm. In sample 3, a light crude oil, the decrease in T_2_ values upon confining into either silica-10 nm or 2.5 nm is nearly 13-fold. The molecules of sample 3 feel the confinement effect independent of pore diameter. In samples 2 and 3, medium-heavy and light crude oils, aliphatics also feel the confinement as indicated by a reduction in T_2(3)_ values of confined crude oils.

Third, the fraction of heavy component (A_1_) is not influenced by confinement in sample 1. The percentage of aliphatic fractions (A_3_) with the largest T_2_ relaxation values gets higher upon confinement in sample 1, a heavy crude oil, while the A_3_ fraction of samples 2 and 3 decreases upon confining into silica-10 and silica-2.5 nm. The A_2_ fraction of sample 1 was assigned to aromatics drops in the confined state. The A_2_ percentages of samples 2 and 3 do not vary significantly in the confined state.

Based on these results, treatments for crude oil production need to be decided appropriately before production. Similar treatments could be applied to produce light and medium-heavy crude oils. The ^o^API gravity borderline value above which the treatment should be switched to heavy crude oil is approximately 30 (Speight, 2002; Riazi, 2005). The results suggest that the SARA analysis of crude oils in bulk needs to be revisited more systematically for the confined crude oils.

**FIGURE 2:**
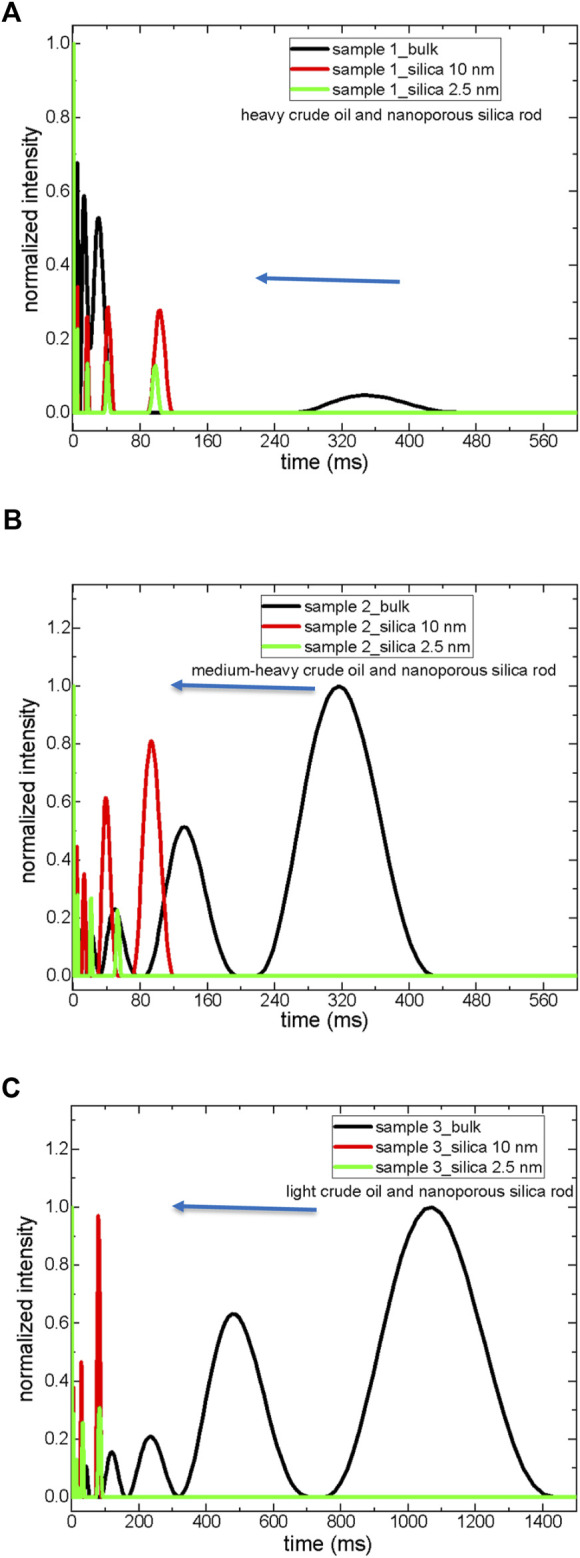
T_2_ distribution curves of crude oils both in bulk and in confined state; sample 1 **(A)**, sample 2 **(B)**, and sample 3 **(C)**.

T_2_ distribution data obtained by Inverse Laplace Transformation (ILT) of T_2_ decaying curves show a systematic trend for all three samples (Figure 2). As the crude oils were confined into silica-10 nm and 2.5 nm, the T_2_ peaks became narrower and shifted towards lower values. Comparison of T_2_ distributions indicates broader T_2_ peaks for sample 3 in bulk compared to samples 1 and 3. This clearly shows the influence of confinement on the dynamics of crude oil molecules. Considering SARA fractions as saturates, aromatics, resins, and asphaltenes, it is possible to say that heavy crude oil has lower intensity aliphatic T_2_ peak. In contrast, the T_2_ peak assigned to aliphatics becomes broader for medium-heavy and light crude oils.

### 3.2 Dynamics of crude oils confined into nanoporous silica rods: T_1_ NMR results

**FIGURE 3:**
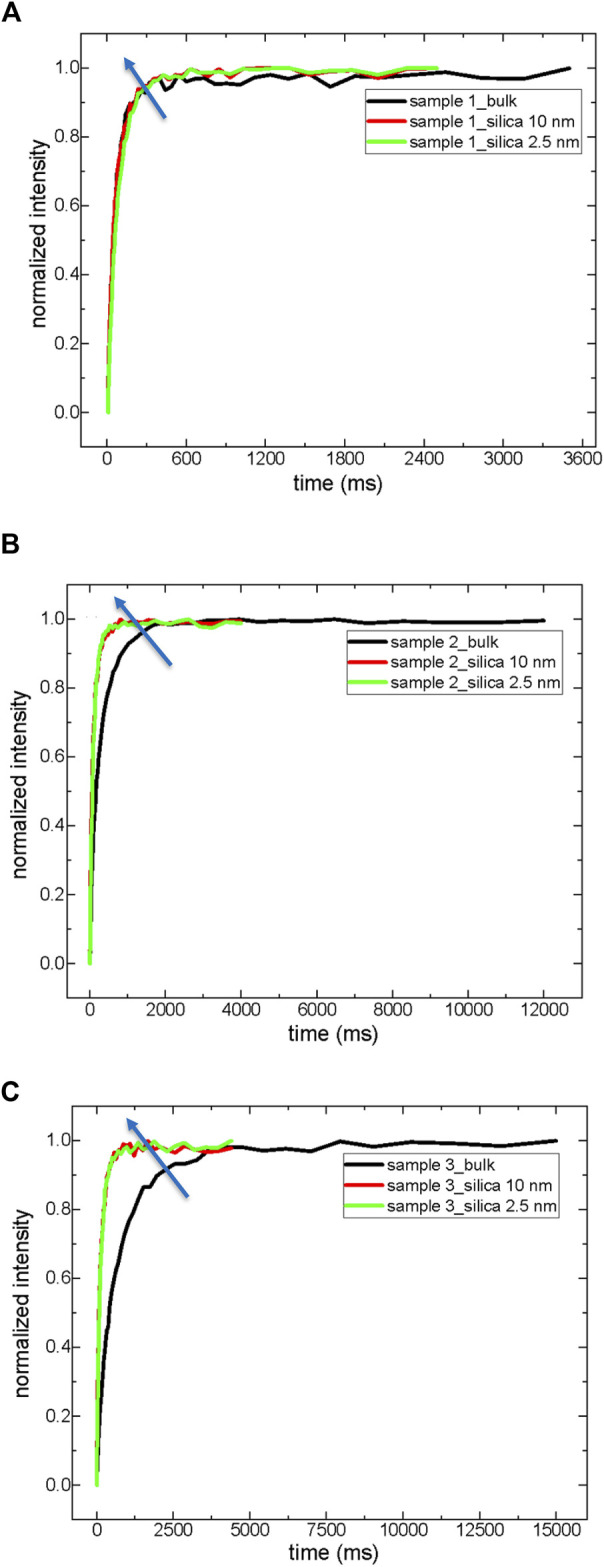
T_1_ relaxation curves of crude oils both in bulk and in a confined state; sample 1 **(A)**, sample 2 **(B)**, and sample 3 **(C)**.

T_1_ is a more sensitive experiment. The T_1_ values are obtained by the exponential growth function shown above (see Eq. 4). Table 5 summarizes the T_1_ results, and Figure 3 compares the T_1_ curves of crude oils in bulk and confined states.

**TABLE 5:** T_1_ relaxation values of crude oils in bulk and confined state. The crude oils are the ones whose physical properties are briefed in Table 1.

T_1_ relaxation	T_1_ (1) (ms)	T_1_ (2) (ms)	T_1_ (3) (ms)	A1%	A2%	A3%
Bulk crude oils						
1	17.2 ± 0.0	60.8 ± 2.6	440.2 ± 0.0	14.0	81.3	4.7
2	53.6 ± 3.6	293.2 ± 16.4	772.8 ± 0.0	26.0	56.7	17.3
3	118.8 ± 21.0	645.2 ± 49.0	2174.4 ± 0.0	17.2	67.7	15.2
Crude oils in silica 10 nm						
1	31.8 ± 4.1	105.6 ± 31.2	264.9 ± 0.0	55.2	40.3	4.5
2	37.8 ± 5.8	86.2 ± 0.0	205.8 ± 35.9	36.9	36.9	26.2
3	13.3 ± 0.0	56.2 ± 9.1	177.4 ± 16.8	2.5	38.3	59.3
Crude oils in silica 2.5 nm						
1	7.6 ± 6.6	71.5 ± 8.7	205.0 ± 69.6	5.4	75.8	18.8
2	7.8 ± 0.0	76.3 ± 8.2	175.1 ± 49.1	1.4	75.0	23.5
3	7.2 ± 0.0	47.5 ± 6.3	156.8 ± 0.0	5.4	27.0	67.6

The crude oils are the ones whose physical properties are briefed in Table 1. Sample 1: heavy crude oil, sample 2: medium heavy crude oil, sample 3: light crude oil.

As expected, T_1_ values in bulk and confined states are longer than corresponding T_2_ values. T_1_ values reflect some physical properties of crude oils, such as ^o^API gravity in bulk. For instance, the T_1_ values of sample 1 with lower ^o^API gravity are much shorter than those of samples 2 and 3 with higher ^o^API gravity. Similar to T_2_ results, T_1_ results in a confined state also deviate from bulk attitude. As in T_2_, the deviation degree of sample 1 (heavy crude oil) in a confined state compared to bulk is lower than that of samples 2 and 3 in a confined state compared to their bulk dynamics. T_1(3)_ of sample 1 becomes only 2.2 times shorter inside silica-2.5 nm nanopores, while T_1(3)_ values of samples 2 and 3 are shortened 4.4 and 13.9 times, respectively, inside silica-2.5 nm nanopores. Surprisingly, the T_1_(1) and T_1_(2) values of sample 1 become longer in silica-10 nm nanopores, then reduced inside silica-2.5 nm nanopores. When the molecules are confined into nanoporous systems in heavy crude oil, the confinement effect suggests thinking beyond the conventional concept of SARA (saturates, aromatics, resins, and asphaltenes). A remarkable portion of saturates and aromatics-resins feel the confinement which in turn causes the molecules of aromatics and resins to act similar to asphaltenes. This is also reflected in A_1_ and A_2_ percentages in sample 1 inside silica-10 nm. A different situation occurs in samples 1 and 2 inside silica-2.5 nm nanopores. Both fractions of saturates-like and resins/aromatics-like fractions increase while the percentage of asphaltenes decreases. In the light crude oil (sample 3) confined into silica-2.5 nm nanopores, the percentage of saturates-like increases, but fractions of resins-aromatics and asphaltenes decrease. These fluctuations in the percentages of different sub-components of crude oils indicate that the conventional SARA approach needs further analysis and better definition for especially confined states of matter.

Light crude oils contain approximately 1% or less than 1% asphaltenes, and medium-heavy crude oils might have a maximum of 3%–4% asphaltenes. On the contrary, the fraction of heavy crude oil asphaltenes might exceed 8%–9% (Volkov et al., 2021). Asphaltenes in the confined state might influence the dynamics of the other components of crude oils (Espinat et al., 2017; Korb et al., 2013). For instance, according to the study by Espinat et al. (2017) on the dynamics of confined crude oil in the porous alumina catalysts by LF-NMR, asphaltene nanoaggregates and assemblies can act as large substances collected in the pores with mobility that affects toluene mobility. When the porous catalyst has macropores, the asphaltene collection with a larger population becomes less influential, permitting faster dynamics of asphaltenes and toluene. Referring to the work by Espinat et al. (2017), the current results suggest a similar explanation.

T_1_ and T_2_ relaxation dynamics of heavy crude oils with a large amount of asphaltenes do not change in the confined state in silica-10 nm (see Tables 4, 5; Figures 1–3). T_1_ and T_2,_ relaxation dynamics of medium-heavy and light crude oils with less fraction of asphaltenes, changed remarkably in silica-10 and silica-2.5 nm rods. Asphaltene nanoaggregates are formed in the sub-regions closer to the walls of the nanopores of silica-10 nm in sample 1 (a heavy crude oil). Nanoaggregates of asphaltenes of sample 1 in silica-10 nm rod result in faster dynamics of the rest of the molecules, such as maltenes, as suggested by Espinat et al. (2017). Nanoaggregate formation and assemblies of asphaltenes of sample 1 “confine” maltene molecules in the pores of the silica-2.5 nm rod. The stronger confinement of maltene molecules in silica-2.5 nm rod is reflected as shorter T_1_ and T_2_ relaxation values than those of crude oil in bulk. For samples 2 and 3, medium-heavy and light crude oils, respectively, reductions in T_1_ and T_2_ values are observed in silica-10 and silica-2.5 nm rods. Since samples 2 and 3 do not contain a high fraction of asphaltenes, the relaxation dynamics of maltene molecules in the nanopores of silica rods observe a severe constraint confinement effect by direct interaction with the pore walls. The above explanation could be summarized as follows: a relaxation model of irregular surface dynamics of maltene molecules near nanoaggregates of asphaltenes and bulk dynamics between clusters of these nanoaggregates where the nanoaggregates are closer to the pore walls in heavy crude oils (Korb et al., 2013). These clusters based on the nanoaggregates are less influential for medium-heavy and light crude oils because of their lower fraction.

The present results also show that a relatively higher fraction of asphaltenes, as in heavy crude oils, influence wettability alteration, defined as making the reservoir rock more water-wet in the petroleum industry (Mohammed and Babadagli, 2015). According to the current results, wettability alteration is an internal factor in heavy crude oil. In other words, nanoaggregate clusters of asphaltenes of heavy crude oil formed in silica-10 nm rod wet the pore walls. On the other hand, in medium-heavy and light crude oils with a lower fraction of asphaltenes, the fraction of asphaltenes is insufficient to form the nanoclusters that might influence the wettability alteration.

The wettability alteration in reservoirs is an essential issue because wettability in the pores of oil reservoirs could be either homogeneous or heterogeneous. In homogeneous wetting, the whole rock surface is wetted uniformly by either water or oil. On the contrary, in heterogeneous wettability, different surface zones show another tendency of wetting regimes for oil or water. Altering the wettability, when there is complete water wetting or heterogeneous wetting by distinct regions, has a direct relationship with enhanced oil recovery processes (Yefei et al., 2011). The method of treating oil reservoirs by different approaches, such as nanofluids and surfactants (Yefei et al., 2011; Eltoum et al., 2021), aims at improving oil recovery and reducing the trapped crude oil in reservoirs. The present results show that in confined crude oils, oil-trapped “isolated globules” could be treated by, at first, understanding the nature of crude oil, especially in a confined state. To provide which conditions could be better, the correlations between the identification of crude oil (heavy, medium-heavy, and light), asphaltenes (the heaviest fraction of crude oils), wettability alteration treatment (nanofluids, the salinity of water, surfactants, etc.), and potential enhancements in oil recovery need to be mapped.

In addition to mapping the correlations mentioned above, a potential issue that might affect the wettability in the current results might arise from the aging of the crude oil samples, as suggested by Medina-Rodriguez et al. (2020). The work of Medina-Rodriguez et al. analyzes glass beads solely with oil and demonstrates the effect aging has on surface wettability by changes in T_2_ distribution and diffusion. The effect of aging is more pronounced in untreated glass beads, with a shift from a peak height of 40.0 ± 0.1 ms at the end of 1 day, attributed to a water-wet situation, to a peak height of 35.7 ± 0.1 ms at the end of 3 weeks, explained by an oil-wet situation. However, in treated glass beads, there was a slight shift from 35.1 ± 0.2 ms to 34.7 ± 0.1 ms as a function of aging (1 day versus 3 weeks). The slight shift was considered to further enhance oleophilicity. Similar to the oil-wetted treated glass beads with 3 weeks of saturation, silica rods were kept long enough in the crude oil samples to reach complete saturation of the tiny nanopores. Therefore, the behaviors of crude oils confined in the nanoporous silica rods are studied in an aging regime closer to the treated glass beads. As a result, oil-wetted nanoporous silica rods were subjected to analysis in the present study.

### 3.3 Dynamics of maltenes confined into nanoporous silica rods: T_2_ and T_1_ NMR results

**FIGURE 4:**
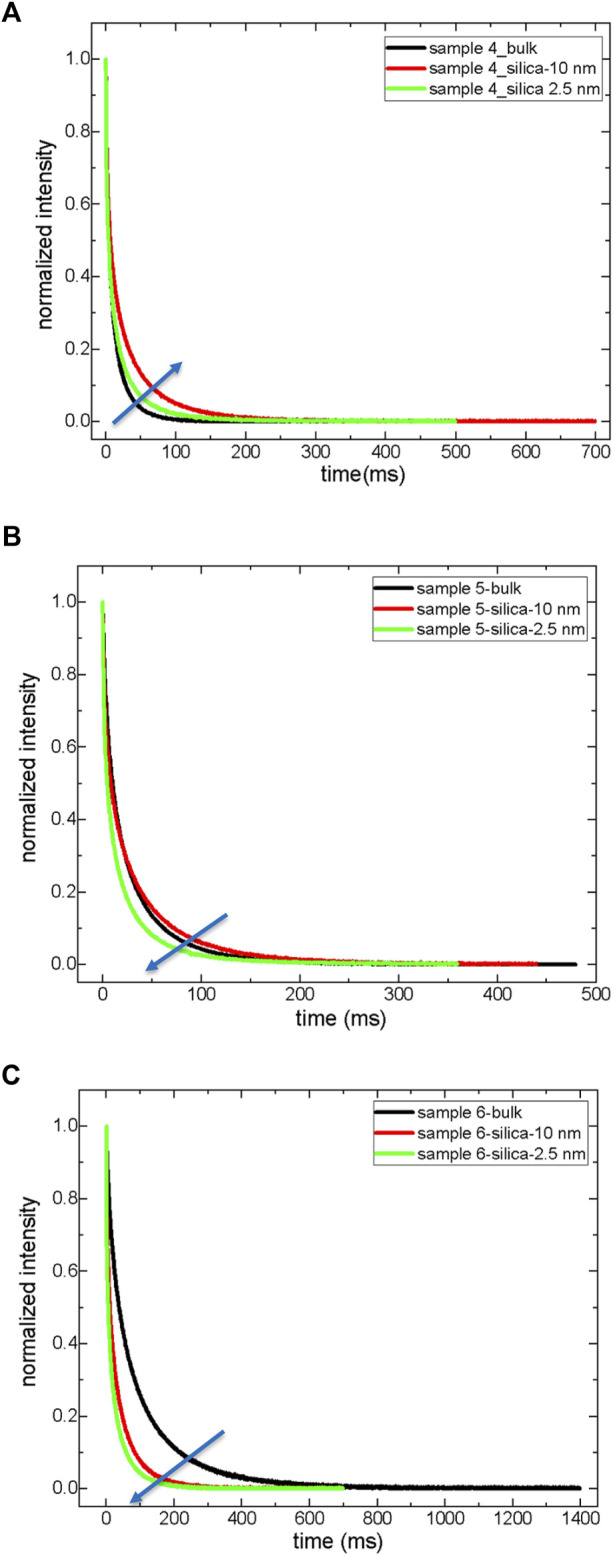
T_2_ relaxation curves of maltenes both in bulk and in confined state; sample 4 **(A)**, sample 5 **(B)**, and sample 6 **(C)**.

**FIGURE 5:**
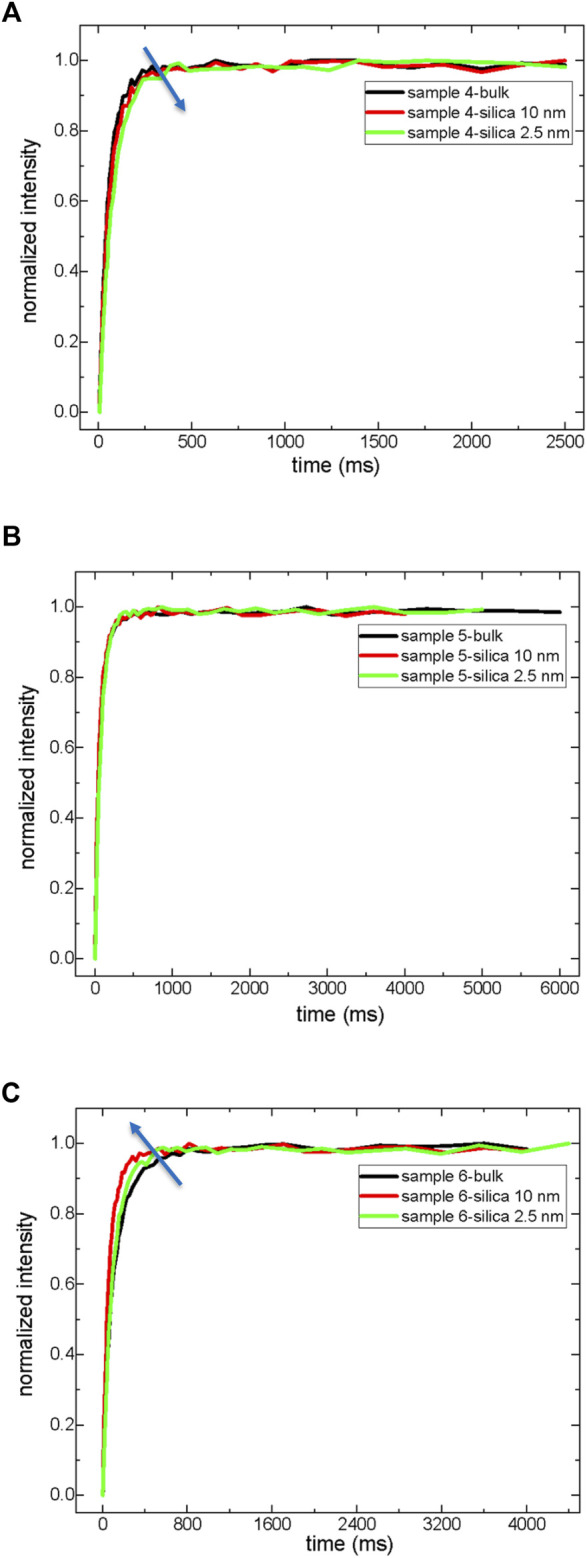
T_1_ relaxation of exponential growth curves of maltenes both in bulk and in a confined state. Sample 4 **(A)**, Sample 5 **(B)**, and Sample 6 **(C)**.

It is not possible to measure the dynamics of asphaltenes in solid-state by low-field NMR relaxometry. However, asphaltenes’ indirect influence on maltenes’ dynamics is NMR measurable. Therefore, the next focus is the analysis of the dynamics of maltenes in bulk and confined states (Figures 4, 5; Tables 6, 7). The first significant result is a reduction in both T_1_ and T_2_ maltenes’ values compared to the corresponding crude oils in bulk. There is also a systematic decrease in both T_1_ and T_2_ values of maltenes in a confined state compared to those of bulk.

**TABLE 6:** T_2_ relaxation values of maltenes in bulk and confined state. The maltenes are the ones whose physical properties are briefed in Table 1.

T_2_ relaxation	T_2_ (1) (ms)	T_2_ (2) (ms)	T_2_ (3) (ms)	A1%	A2%	A3%
Bulk maltenes						
4	1.3 ± 0.1	6.8 ± 0.2	23.3 ± 0.3	26.1	45.0	28.8
5	2.7 ± 0.1	14.1 ± 0.3	47.3 ± 0.4	24.0	42.3	33.7
6	10.2 ± 0.2	50.1 ± 0.5	156.3 ± 0.7	19.0	43.0	38.0
Maltenes in silica 10 nm						
4	1.8 ± 0.0	13.6 ± 0.2	56.5 ± 0.4	38.0	35.2	26.9
5	1.8 ± 0.0	14.3 ± 0.3	57.3 ± 0.5	34.0	34.0	32.1
6	1.9 ± 0.0	15.7 ± 0.2	60.7 ± 0.3	29.5	32.4	38.1
Maltenes in silica 2.5 nm						
4	1.4 ± 0.0	10.2 ± 0.2	40.6 ± 0.5	43.0	20.2	36.8
5	1.3 ± 0.0	9.8 ± 0.2	39.7 ± 0.5	40.4	36.0	23.7
6	1.5 ± 0.0	11.8 ± 0.2	49.9 ± 0.3	38.2	32.7	29.1

**TABLE 7:** T_1_ relaxation values of maltenes in bulk and confined state. The maltenes are the ones whose physical properties are briefed in Table 1.

T_1_ relaxation	T_1_ relaxation	T_1_ (2) (ms)	T_1_ (3) (ms)	A1%	A2%	A3%
Bulk maltenes						
4	34.1 ± 1.3	71.8 ± 6.9	201.3 ± 0.0	59.6	36.3	4.0
5	30.0 ± 5.3	75.8 ± 9.4	258.6 ± 0.0	33.5	60.8	5.7
6	22.9 ± 4.5	95.7 ± 12.9	306.6 ± 65.3	18.4	63.3	18.4
Maltenes in silica 10 nm						
4	30.4 ± 5.7	59.8 ± 0.0	158.4 ± 0.0	36.1	50.5	13.4
5	28.8 ± 2.9	68.9 ± 0.0	160.7 ± 0.0	38.3	50.7	11.0
6	38.8 ± 1.0	74.5 ± 0.0	168.3 ± 0.0	63.9	20.5	15.6
Maltenes in silica 2.5 nm						
4	10.2 ± 0.0	68.5 ± 4.4	143.6 ± 0.0	5.1	83.3	11.7
5	10.3 ± 0.0	66.6 ± 2.1	148.5 ± 0.0	2.2	84.3	13.4
6	11.4 ± 0.0	73.0 ± 10.2	154.7 ± 41.0	2.9	64.9	32.2

The confinement effect is more pronounced in maltenes (sample 6) of light crude oil (sample 3). The fractional analysis by the A_1_, A_2_, and A_3_ components of T_2_ does not reflect significant changes. In samples 4 and 5 inside nanopores of silica-2.5 nm rod, the percentages of resins decrease, and resin molecules act similarly to the aromatics. In sample 6, maltenes of light crude oil, while the fraction of resins decreases, the saturates’ percentage increases. This was also observed in the light crude oil (sample 3) inside nanopores of the silica-2.5 nm rod. These results clearly show that resin molecules might indicate similar attitudes to saturates in natural confinement with pores in different scales ranging from nano to micro and even macro, while aromatics might show resin-like behaviors. That means in addition to saturates, aromatics, resins, and asphaltenes. There could be aromatics-to-resins and resins-to-saturates as sub-groups in crude oil. Such sub-groups might affect wettability alterations of crude oils in rock cores. Therefore, these issues must be considered before deciding on proper treatments for crude oil production.

### 3.4 Dynamics of crude oils confined into nanoporous silica powder: T_2_ and T_1_ NMR results

**FIGURE 6:**
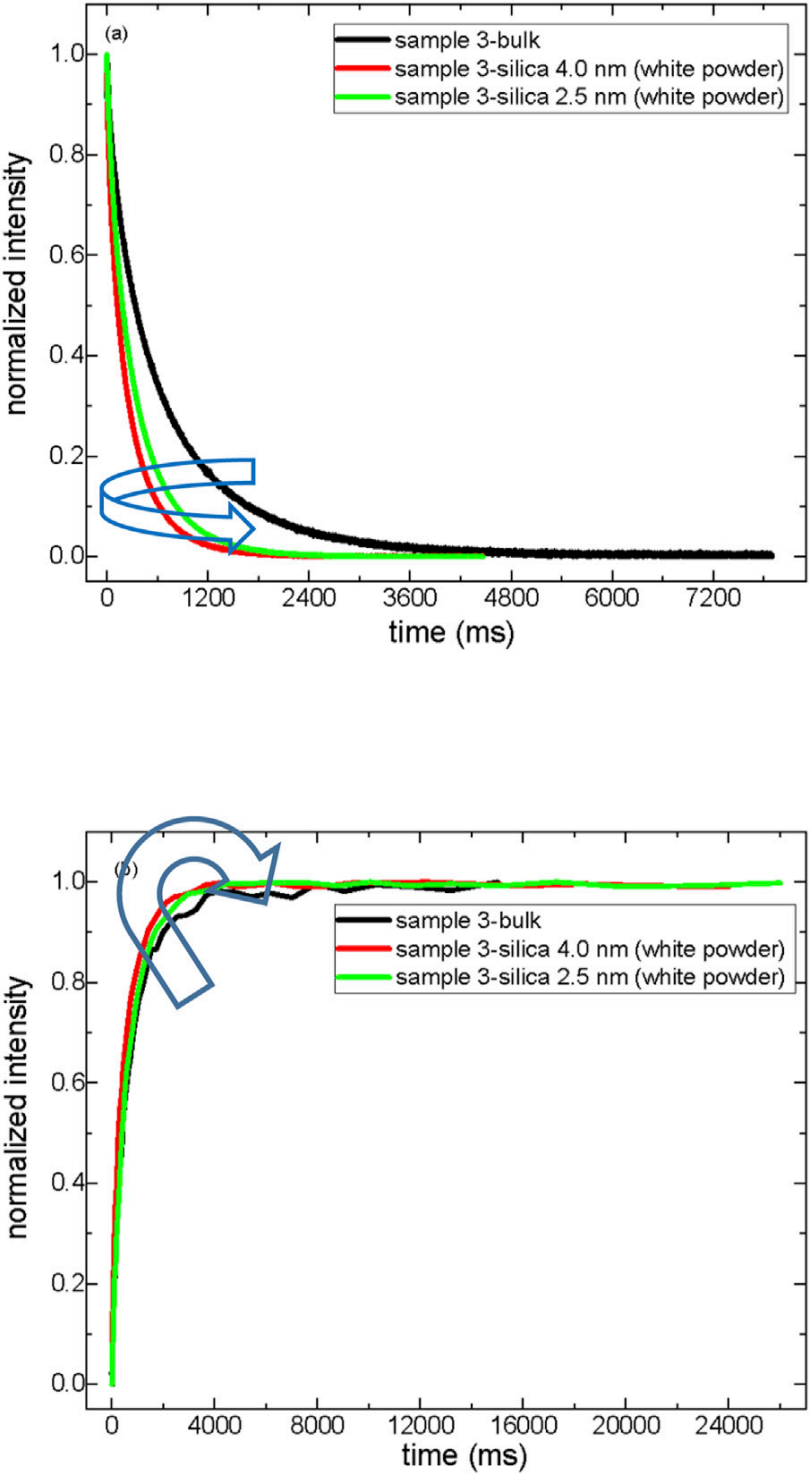
**(A,B)** T_2_ relaxation curves of sample 3 both in bulk and in confined state **(A)**. T_1_ relaxation exponential growth curves of sample 3 both in bulk and in confined state **(B)**.

The dynamical behaviors of sample 3-a light crude oil whose physical properties are shown in Table 1-inside the nanopores of white powder samples of silica-4.0 nm and silica-2.5 nm are studied. The primary goal, in this case, is to compare the effect of pore diameter and S/V ratio on the dynamics of light crude oil (see Table 2 of Ok et al. (2020) of listing the physical properties of the two silica materials). Table 8 lists the T_1_ and T_2_ values of sample 3 in bulk and a confined state in white powder nanoporous silica samples, while Figures 6A, B displays the comparisons of T_2_ and T_1_ relaxation.

**TABLE 8:** T_2_ and T_1_ relaxation values of sample 3 in bulk and confined state. The physical properties of sample 3 are briefed in Table 1.

T_2_ relaxation	T_2_ (1) (ms)	T_2_ (2) (ms)	T_2_ (3) (ms)	A1%	A2%	A3%
Sample 3	78.7 ± 2.0	378.4 ± 4.4	1030.0 ± 4.0	10.6	39.4	50.0
200 mg silica_4.0 nm + 4.0 ml sample 3	16.9 ± 0.2	141.9 ± 0.9	386.2 ± 0.9	13.4	37.1	49.5
100 mg silica_2.5 nm + 4.0 ml sample 3	37.9 ± 0.5	177.4 ± 1.4	457.6 ± 0.71	10.1	30.3	59.6

T_1_ relaxation	T_1_ (1) (ms)	T_1_ (2) (ms)	T_1_ (3) (ms)	A1%	A2%	A3%
Sample 3	118.8 ± 24.9	645.2 ± 104.8	2174.4 ± 987.2	16.7	68.0	15.2
200 mg silica_4.0 nm + 4.0 ml sample 3	41.8 ± 7.5	213.1 ± 28.8	785.7 ± 32.5	10.9	34.1	55.0
100 mg silica_2.5 nm + 4.0 ml sample 3	49.8 ± 14.9	301.1 ± 34.0	907.5 ± 33.8	4.1	34.7	61.2

Chemically speaking, the two white powdered silica samples are provided as SiO_2_, but it was already shown that they are H_2_O absorbing systems, and their pore walls are decorated with −OH groups (Ok et al., 2017). Thus, nanoporous SiO_2_(−OH) matrixes are ideal enough to resemble geologically relevant sub-surfaces. As shown in Table 2 of Ok et al. (2020), the silica-2.5 nm material has a relatively low pore volume but has the highest pore surface area due to its nearly micropore feature and mesoporous structures than the silica-4.0 nm probe. The surface-to-volume ratio (S/V) demonstrates that a 2.5 nm pore diameter silica has a higher S/V value. This ratio will be used as a parameter in the following discussion. As listed in Table 8, the T_2_ and T_1_ values of confined crude oil inside the nanoporous systems are lower than those in bulk. However, the T_2_ and T_1_ values of crude oil in silica-4.0 nm are lower than those in silica-2.5 nm. This shows that the dynamics of crude oil in the confined state do not change as a function of pore diameter and instead by S/V ratio. Timur claimed that in a three-component NMR model, the pore spaces of a porous medium were divided into three groups based on their S/V ratio distribution (Timur, 1969). He explained that the longer T_1_ times correspond to the smaller S/V ratios and the larger pores. T_1_ times become longer when sample 3 molecules are confined into silica-2.5 nm with the highest S/V ratio, while the shortest T_1_ times are observed upon confining H_2_O molecules into silica-4.0 nm with the lowest S/V ratio.

For this reason, the trend in T_1_ times of sample 3 confined into silica materials is attributed to the S/V ratios rather than pore diameter. These measurements were conducted by keeping the pore volume of each nanoporous silica constant at around 120 cm^3^ (see Table 2 of Ok et al. (2020)). When the pore volume is kept constant at approximately 120 cm^3^, water has the longest T_1_ value in the mixture with silica-2.5 nm. Similarly, the highest T_2_ was obtained for the same crude oil and silica-2.5 nm mixture.

When the pore diameter is enormous and the S/V ratio is small, fluid molecules first wet the surface of the pore walls. Further filling happens smoothly from the pore wall toward the center of the pore. Then complete filling of the pores is achieved (Grünberg et al., 2004). In the case of nanoporous silica with a small pore diameter and large S/V ratio, the filling mechanism occurs differently: first, the center is filled; hence there is a coexistence of filled pore segments with wetted pores. Further filling of the pores grows axially in the direction of the pore axis. The pore volume is also related to pore length when cylindrical pores are assumed. The nanoporous silica with a small pore diameter and long pore axis leads to larger pore volumes. The long pore length (large pore volume) gives enough freedom for confined crude oil molecules. Relatively free confined crude oil molecules prefer motion in the axial direction, resulting in weaker interactions with pore walls. This is a possible explanation of larger T_1_ values for pores with smaller pore diameters and larger pore volumes.

The last set of experiments conducted included the confinement of crude oil with ^o^API gravity of 35.92 and density of 0.83707 g/cm^3^ at 25°C and brine into powdered silica-4.0 nm and silica-2.5 nm matrixes. The results are shown in Table 9. The shorter relaxation values of light crude oil arise from the heavy components such as asphaltene and resins. In contrast, the longer relaxation values are assigned to the lighter fractions, including saturates and aromatics (Ok et al., 2021).

**TABLE 9:** T_2_ and T_1_ relaxation values of light crude oil (API gravity of 35.92 and density of 0.83707 g/cm^3^ at 25°C) and brine in bulk and confined state.

Sample	T_2_ (1) (ms)	T_2_ (2) (ms)	T_2_ (3) (ms)	T_1_ (1) (ms)	T_1_ (2) (ms)	T_1_ (3) (ms)
Pure brine (250,000 ppm)	2604.8 ± 0.2			2900.0 ± 100.0		
200 mg silica-4.0 nm + 0.4 ml brine	17.6 ± 0.3	4.5 ± 0.6		1839.3 ± 11.3		
100 mg silica-2.5 nm + 0.4 ml brine	125.1 ± 1.0	26.3 ± 0.2		1852.9 ± 9.4		
Light crude oil	839.4 ± 0.9	210.9 ± 0.7		945.7 ± 24.1	225.0 ± 0.0	
200 mg silica-4.0 nm + 0.4 ml light crude oil	164.3 ± 0.7	38.2 ± 0.4		431.5 ± 9.6	77.9 ± 16.7	
100 mg silica-2.5 nm + 0.4 ml light crude oil	414.3 ± 0.8	103.9 ± 0.7		736.0 ± 26.1	177.8 ± 14.6	
200 mg silica-4.0 nm + 0.32 ml light crude oil +0.08 ml brine	189.3 ± 1.2	57.0 ± 0.6	7.1 ± 0.2	604.5 ± 36.9	210.7 ± 29.3	29.0 ± 22.8
100 mg silica-2.5 nm + 0.32 ml light crude oil +0.08 ml brine	520.7 ± 1.5	190.5 ± 1.4	26.8 ± 0.2	1059.3 ± 47.5	332.3 ± 24.9	44.7 ± 7.4
200 mg silica-4.0 nm + 0.28 ml light crude oil +0.12 ml brine	283.1 ± 2.2	78.8 ± 0.9	13.5 ± 0.2	976.1 ± 66.0	295.5 ± 29.7	57.2 ± 16.0
100 mg silica-2.5 nm + 0.28 ml light crude oil +0.12 ml brine	524.7 ± 2.2	177.0 ± 2.1	25.2 ± 0.3	1161.3 ± 103.6	374.7 ± 63.3	63.7 ± 15.7

Brine: prepared using NaCl, CaCl_2_, and MgCl_2_ with a concentration of 250.000 ppm; light crude oil. ^o^API, gravity of 35.92 and density of 0.83707 g/cm3 at 25°C (light crude oil is sample 4 in Ok et al., (2019)).

The effect of confinement is more pronounced for brine, according to the T_2_ results. More importantly, both fluids’ relaxation values, both T_2_ and T_1_, are higher when the fluids are confined into silica-2.5 nm. Three-component fittings evaluate the relaxation values when both fluids are confined together in the nanoporous proxies. T_2_(3) and T_1_(3), the shortest relaxation values, are assigned to brine, while T_2_(1) and T_1_(1), the longest relaxation values, are attributed to the mixture of brine and crude oil, and T_2_(2) and T_1_(2) to mainly crude oil. A portion of brine forms an interphase layer with pore walls; this might help enhance crude oil recovery if, for example, brine is enriched with inorganic nanoparticles such as ZnO (Alomair et al., 2022). Brine fills the nanopores faster than crude oil molecules due to its lower viscosity and forms a layered structure *via* interaction with −OH decorating the pore walls. Hence, the free space in the pore volume is decreased, and less crude oil is confined into the nanoporous silica proxies. This is reflected in longer relaxation values, T_2_(1), T_2_(2), T_1_(1), and T_1_(2), when both fluids were confined than when either of the fluids was confined separately. The results of the last set of samples can be related to wettability alteration. The intense interaction between water molecules and the pore walls shows the possibility of wettability alteration, referring to the process of making the reservoir rock more water-wet (Mohammed and Babadagli, 2015) by a facile approach of utilizing brine.

## 4 Conclusion

A substantial deviation is observed in the dynamics of crude oil in the confined state compared to bulk. Mathematical analysis of NMR relaxation curves of confined and bulk crude oils with different fractions of SARA (saturates, aromatics, resins, asphaltenes) and with maltenes without asphaltenes indicate that the conventional SARA approach needs a better definition for the especially confined state of matter. The NMR relaxation behavior of maltenes shows that in natural confinement with pores in different scales ranging from nano to micro, macro resin molecules might act like saturates, or aromatics might show resin-like behaviors. Confinement of brine and a light crude oil into white powdered nanoporous silica proxies demonstrates that brine could be utilized along with some additives such as nanoparticles for oil recovery. Based on the analysis of the dynamical behaviors of confined crude oils, treatments for medium-heavy and light crude oil productions might have similarities. The sub-groups of aromatics-to-resins and resins-to-saturates might influence wettability alterations of crude oils in rock cores. Therefore, these issues must be considered before deciding on proper crude oil production and enhanced oil recovery treatments. The current results on T_1_ and T_2_ relaxation dynamics of confined heavy crude oils suggest that the nanoaggregate formation of asphaltenes allow faster dynamics of maltene molecules, especially in relatively larger pores, such as in the pores of silica-10 nm rods. Hence, the nanoaggregate formation of asphaltenes contributes to wettability alteration. In addition to treating the nanoclusters of asphaltene aggregates in heavy crude oil confined as “isolated globules,” as explained above, brine might be utilized for wettability alteration in enhanced oil recovery studies. The current results resume that the intrinsic properties of crude oils, including viscosity arising from heavy components, such as asphaltenes, might be used for enhanced oil recovery studies.

## Data Availability

The raw data supporting the conclusion of this article will be made available by the author, without undue reservation.

## References

[B1] Alomair O. ElSharkawy A. Al-Bazzaz W. Ok S. (2022). Low-field NMR investigation on interaction of ZnO nanoparticles with reservoir fluids and sandstone rocks for enhanced oil recovery. J. Pet. Expl. Prod. Technol. 10.1007/s13202-022-01547-5

[B2] Anovitz L. M. Cole D. R. (2015). Characterization and analysis of porosity and pore structures. Rev. Mineral. Geochem. 80, 61–164. 10.2138/rmg.2015.80.04

[B3] Canan T. F. Ok S. Al-Bazzaz W. Ponnuswamy S. Fernandes M. Al-Shamali M. (2022). Rapid characterization of crude oil by NMR relaxation using new user-friendly software. Fuel 320, 123793–123801. –7. 10.1016/j.fuel.2022.123793

[B4] Coates G. R. Xiao L. Prammer M. G. (1999). NMR logging principles and applications. Houston: Haliburton Energy Services, 1–233.

[B5] Cole D. R. Gruszkiewicz M. S. Simonson J. M. Chialvo A. A. Melnichenko Y. B. (2004). “Influence of nanoscale porosity on fluid behavior,” in Water-rock interaction. Editors Wanty R. Seal R. (Berlin, Germany: Springer), Vol. 1, 735–739.

[B6] Cole D. R. Ok S. Striolo A. Phan A. (2013). Hydrocarbon behavior at nanoscale interfaces. Rev. Mineral. Geochem. 75, 495–545. 10.2138/rmg.2013.75.16

[B7] da Silva P. N. Goncalves E. C. Rios E. H. Muhammad A. Moss A. Pritchard T. (2015). Automatic classification of carbonate rocks permeability from ^1^H-NMR relaxation data. Expert sys. Appl. 42, 4299–4309. 10.1016/j.eswa.2015.01.034

[B8] de Almeida J. M. Miranda C. R. (2016). Improved oil recovery in nanopores: NanoIOR. Sci. Rep. 6, 28128. 10.1038/srep28128 27319357 PMC4913300

[B9] Dvoyashkin M. Valiullin R. Kärger J. Einicke W.-D. Glaser R. (2007). Direct assessment of transport properties of supercritical fluids confined to nanopores. J. Am. Chem. Soc. 129, 10344–10345. 10.1021/ja074101+ 17672467

[B10] Dvoyashkin N. K. Filipov A. (2018). Diffusivity of crude oils contained in macroporous medium: ^1^H NMR study. Mendeleev Comm. 28, 222–224. 10.1016/j.mencom.2018.03.039

[B11] Eltoum H. Yang Y.-L. Hou J.-R. (2021). The effect of nanoparticles on reservoir wettability alteration: A critical review. Pet. Sci. 18, 136–153. 10.1007/s12182-020-00496-0

[B12] Espinat D. Gaulier F. Norrant F. Barbier J. Guichard B. Rivallan M. (2017). Characterization of asphaltenes in solution and inside the pores of catalysts by ^1^H NMR relaxometry. Energy fuels. 31, 7382–7395. 10.1021/acs.energyfuels.7b00139

[B13] Freedman R. Heaton N. (2004). Fluid characterization using nuclear magnetic resonance logging. Petrophys 46, 241–250.

[B14] Gautam S. S. Ok S. Cole D. R. (2017). Structure and dynamics of confined C-O-H fluids relevant to the subsurface: Application of magnetic resonance, neutron scattering, and molecular dynamics simulations. Front. Earth Sci. 5, 43–51. –19. 10.3389/feart.2017.00043

[B15] Gelb L. D. Gubbins K. E. Radhakrishnan R. Sliwinska-Bartkowiak M. (1999). Phase separation in confined systems. Rep. Prog. Phys. 62, 1573–1659. 10.1088/0034-4885/62/12/201

[B16] Grünberg B. Emmler T. Gedat E. Shenderovich I. Findenegg G. H. Limbach H.-H. (2004). Hydrogen bonding of water confined in mesoporous silica MCM-41 and SBA-15 studied by 1H solid-state NMR. Chem. Eur. J. 10, 5689–5696. 10.1002/chem.200400351 15470692

[B17] Kashif M. Cao Y. Yuan G. Asif M. Javed K. Mendez J. N. (2019). Pore size distribution, their geometry and connectivity in deeply buried Paleogene Es1 sandstone reservoir, Nanpu Sag, East China. Pet. Sci. 16, 981–1000. 10.1007/s12182-019-00375-3

[B18] Korb J.-P. Louis-Joseph A. Benamsili L. (2013). Probing structure and dynamics of bulk and confined crude oils by multiscale NMR spectroscopy, diffusometry, and relaxometry. J. Phys. Chem. B 117, 7002–7014. 10.1021/jp311910t 23687962

[B19] Liebscher A. Heinrich C. A. (Editors) (2007). “Fluid-fluid interactions,” Reviews in mineralogy and geochemistry (Chantilly VA: Mineralogical Society of America), 65.

[B20] Liu K.-H. Zhang Y. Lee J.-J. Chen C.-C. Yeh Y.-Q. Chen S.-H. (2013). Density and anomalous thermal expansion of deeply cooled water confined in mesoporous silica investigated by synchrotron X-ray diffraction. J. Chem. Phys. 139, 064502. 10.1063/1.4817186 23947866

[B21] Liu L. Chen S. H. Faraone A. Yen C. W. Mou C. Y. Kolesnikov A. I. (2006). Quasielastic and inelastic neutron scattering investigation of fragile-to-strong crossover in deeply supercooled water confined in nanoporous silica matrices. J. Phys. Condens. Matter 18, S2261–S2284. 10.1088/0953-8984/18/36/s03

[B22] Lyu C. Ning Z. Wang Q. Chen M. (2018). Application of NMR T_2_ to pore size distribution and movable fluid distribution in tight sandstones. Energy & Fuels 32, 1395–1405. 10.1021/acs.energyfuels.7b03431

[B23] Majumdar R. D. Gerken M. Mikula R. Hazendonk P. (2013). Validation of the Yen–Mullins model of athabasca oil-sands asphaltenes using solution-state 1H NMR relaxation and 2D HSQC spectroscopy. Energy fuels. 27, 6528–6537. 10.1021/ef401412w

[B24] Medina-Rodriguez B. X. Reilly T. Wang H. Smith E. R. Garcia-Olvera G. Alvarado V. (2020). Time-domain nuclear magnetic resonance determination of wettability alteration: Analysis for low-salinity water. Appl. Sci. 10, 1017. 10.3390/app10031017

[B25] Mohammed M. Babadagli T. (2015). Wettability alteration: A comprehensive review of materials/methods and testing the selected ones on heavy-oil containing oil-wet systems. Adv. Coll. Inter. Sci. 220, 54–77. 10.1016/j.cis.2015.02.006 25798909

[B26] Mullins O. C. (2011). The asphaltenes. Annu. Rev. Anal. Chem. 4, 393–418. 10.1146/annurev-anchem-061010-113849 21689047

[B27] Ok S. Hoyt D. W. Andersen A. Sheets J. Welch S. A. Cole D. R. (2017). Surface interactions and confinement of methane: A high pressure magic angle spinning NMR and computational chemistry study. Langmuir 33, 1359–1367. 10.1021/acs.langmuir.6b03590 28099024

[B28] Ok S. Hwang B. Liu T. Welch S. Sheets J. M. Cole D. R. (2020). Fluid behavior in nanoporous silica. Front. Chem. 8, 734–741. –20. 10.3389/fchem.2020.00734 33005606 PMC7485247

[B29] Ok S. Mahmoodinia M. Rajasekaran N. Sabti M. A. Lervik A. van Erp T. S. (2019). Molecular structure and solubility determination of asphaltenes. Energy fuels. 33, 8259–8270. 10.1021/acs.energyfuels.9b01737

[B30] Ok S. Sheets J. Welch S. A. Cole D. R. Berman M. Rua A. (2021). High-temperature and high-pressure NMR investigations of low viscous fluids confined in mesoporous systems. Zeitsch. Phys. Chem. 235, 931–959. 10.1515/zpch-2019-1510

[B31] Provencher S. W. (1982). A constrained regularization method for inverting data represented by linear algebraic or integral equations. Comput. Phys. Commun. 27, 213–227. 10.1016/0010-4655(82)90173-4

[B32] Riazi M. R. (2005). Characterization and properties of petroleum fractions. West Conshohocken, PA: ASTM international standards worldwide.

[B33] Song Y.-Q. Kausik R. (2012). NMR application in unconventional shale reservoirs – a new porous media research frontier. Prog. Nuc. Magn. Reson. Spectros. 112–113, 17–33. 10.1016/j.pnmrs.2019.03.002 31481157

[B34] Speight J. G. (2002). Handbook of petroleum product analysis. New York: Wiley-Interscience, 36–39.

[B35] Taborda E. A. Franco C. A. Ruiz M. A. Alvarado V. Cortes F. B. (2017). Experimental and theoretical study of viscosity reduction in heavy crude oils by addition of nanoparticles. Energy fuels. 31, 1329–1338. 10.1021/acs.energyfuels.6b02686

[B36] Timur A. (1969). Pulsed nuclear magnetic resonance studies of porosity, movable fluid, and permeability of sandstones. J. Pet. Technol. 21, 775–786. 10.2118/2045-pa

[B37] Vogel M. (2010). NMR studies on simple liquids in confinement. Eur. Phys. J. 189, 47–64. 10.1140/epjst/e2010-01309-9

[B38] Volkov V. Y. Al-Muntaser A. A. Varfolomeev M. A. Khasanova N. M. Boris V. Sakharov B. V. (2021). Low-field NMR-relaxometry as fast and simple technique for *in-situ* determination of SARA-composition of crude oils. J. Pet. Sci. Eng. 196, 107990. 10.1016/j.petrol.2020.107990

[B39] Walbrecker J. O. Behroozmand A. A. (2012). Surface-NMR measurements of the longitudinal relaxation time T_1_ in a homogeneous sand aquifer in Skive, Denmark. J. Appl. Geophys. 87, 46–52. 10.1016/j.jappgeo.2012.08.009

[B40] Wang X. Xiao S. Zhang Z. He J. (2018). Displacement of nanofluids in silica nanopores: Influenced by wettability of nanoparticles and oil components. Environ. Sci. Nano 5, 2641–2650. 10.1039/c8en00704g

[B41] Westphal H. Surholt I. Kiesl C. Thern H. F. Kruspe T. (2005). NMR measurements in carbonate rocks: Problems and an approach to a solution. Pure Appl. Geophys. 162, 549–570. 10.1007/s00024-004-2621-3

[B42] Xu M. Harris K. D. M. Thomas J. M. Vaughan D. E. W. (2007). Probing the evolution of adsorption on nanoporous solids by *in situ* solid-state NMR spectroscopy. Chem. Phys. Chem. 8, 1311–1313. 10.1002/cphc.200700218 17520588

[B43] Yefei W. Huaimin X. Weizhao Y. Baojun B. Xinwang S. Jichao Z. (2011). Surfactant induced reservoir wettability alteration: Recent theoretical and experimental advances in enhanced oil recovery. Pet. Sci. 8, 463–476. 10.1007/s12182-011-0164-7

